# Death by austerity? The impact of cost containment on avoidable mortality in Italy

**DOI:** 10.1002/hec.4147

**Published:** 2020-08-17

**Authors:** Emanuele Arcà, Francesco Principe, Eddy Van Doorslaer

**Affiliations:** ^1^ Erasmus School of Health Policy & Management Erasmus University Rotterdam Rotterdam The Netherlands; ^2^ Erasmus School of Economics Erasmus University Rotterdam Rotterdam The Netherlands

**Keywords:** avoidable mortality, healthcare expenditure, recovery plan

## Abstract

Does austerity in health care affect health and healthcare outcomes? We examine the intended and unintended effects of the Italian austerity policy Piano di Rientro aimed at containing the cost of the healthcare sector. Using an instrumental variable strategy that exploits the temporal and geographical variation induced by the policy rollout, we find that the policy was successful in alleviating deficits by reducing expenditure, mainly in the southern regions, but also resulted in a 3% rise in avoidable deaths among both men and women, a reduction in hospital capacity and a rise in south‐to‐north patient migration. These findings suggest that—even in a high‐income country with relatively low avoidable mortality like Italy—spending cuts can hurt survival.

## 1 INTRODUCTION

While in most industrialized countries expenditures on health care have kept rising for decades, both in absolute level and as a share of gross domestic product (GDP), estimates of the marginal health benefits of health care spending vary widely between studies, not only because they often refer to very different times and places, but also because they rely on different methodologies. Most have focused on attempting to estimate the marginal health gains of additional spending. In this study, we add to the much smaller literature on the health effect of spending cuts. Most studies that examined the effect of austerity policies implemented after the Great Recession of 2008 had difficulties constructing a counterfactual because generally the entire country population was affected.

A large literature has addressed the relationship between healthcare spending and mortality in an attempt to identify the marginal health effects of additional healthcare spending. Much of this literature (e.g., Bokhari, Gai, & Gottret, 2007; Moreno‐Serra & Smith, 2015) has exploited cross‐national variation and has adopted a variety of methods to try to identify causal effects. A meta‐analysis by Gallet and Doucouliagos (2017) combining estimates from 629 studies worldwide estimated the spending elasticity for mortality to be around −0.13 (for another recent review, see e.g., Nakamura et al., 2016). Other studies have used subnational regional panel data to estimate health effects of public health spending in two‐way fixed effects regression models. A recent example for Italy is Golinelli et al. (2017) who run a two‐way fixed effects model on a panel of 15 years (1999–2013) for 20 Italian regions. They aim to explain the variation in total (not amenable) mortality with a large number of explanatory variables, including breakdown of public health expenditure into seven expenditure components. Only for the largest component (direct spending on health‐related goods and services by the National Health Care Service—i.e., mostly hospital spending), they estimate a significantly negative coefficient. As they do not attempt to use the Piano di Rientro (PdR) reform as an instrumental variable (IV), they simply conclude that higher regional health spending was associated with lower mortality. Borra, Pons‐Pons, and Vilar‐Rodriguez (2020) is another example of an approach that is similar to ours as it employs regional Spanish data and two‐way fixed effects models to estimate the effects, not of health expenditure but of healthcare inputs like the number of hospital workers and beds, and so forth, on disease‐specific mortality rates. But while their period of analysis (1996–2015) includes periods of austerity, they do not explicitly employ health‐specific austerity measures as instruments to estimate causal effects.

A number of studies using regional data have employed IV methods with the aim of obtaining causal estimates of the mortality effects of health spending. Subnational data have the advantage of relying on less heterogeneous measurement of the crucial mortality and expenditure variables that cross‐national data. Some studies have used cross‐sectional data. Martin, Rice, and Smith (2008, 2012) have used cross sections of budgeting data of 295 English Primary Care Trusts to model the link between health spending and health outcomes of various specific programs of care. They report the estimates of the cost of a life year saved for various disease categories, but the validity of the instruments that they used (i.e., the percentage of lone pensioner households and the percentage of the population providing unpaid care) can easily be criticized.

More recently, Edney, Afzali, Cheng, and Karnon (2018) used an IV approach with local statistical area cross‐sectional data to account for endogeneity of healthcare spending to health outcomes in Australia. Like Martin et al. (2008, 2012), they employ the proportion of the population providing unpaid care as the instrument. Their results suggest that, based on the conditional mean, a 1% increase in public health spending was associated with a 2.2% reduction in the number of standardized years of life lost. Andrews, Elamin, Hall, Kyriakoulis, and Sutton (2017) have revisited these estimates for England using other (Generalized method of moments) estimation methods as well as different instruments, based on the “funding rules of the National Health System (NHS)”. Using data on 152 primary care trusts for 2005/2006, they estimate a fairly high elasticity of −0.705 of overall mortality to health expenditure. Claxton et al. (2018) have compared the two different IV approaches used on English data and applied them to both overall mortality and disease‐specific mortality data. Their all‐cause mortality elasticity estimate (−01.09) even exceeds the Andrews et al.’s (2017) estimate. However, as all of these studies had to rely on cross‐sectional data, they can only identify this effect on the basis of between‐area variation, not any variation over time.

Recent subnational studies have used IV methods on panel data. Using essentially the same IVs as Martin, Rice, and Smith (2012), Lomas et al. (2018) used panel data from local health authorities to estimate the marginal productivity of the English NHS over the period 2003–2012. However, since they are interested in obtaining disease‐specific cost per Quality Adjusted Life Years (QALY) estimates and compare these across years and diseases, they do not estimate a fixed effects regression on the 10‐year panel data but estimate year‐specific equations. It is therefore not a surprise that their all‐cause mortality elasticity estimates are similar to those obtained by Andrews et al. (2017) and Claxton et al. (2018).

Finally, Vallejo‐Torres, García‐Lorenzo, and Serrano‐Aguilar (2018) exploited variation across 17 regional health services in Spain and the exogenous changes in expenditure that took place as a consequence of the economic crisis over 5 years of data. They estimated a fixed effects model with an IV approach, using the percentage of the total regional expenditure allocated to health as their main instrument, in a period of austerity but do not use any austerity measures as instruments. They found that health expenditure has a positive and significant effect on population health, with an average spending elasticity estimate of the population Quality‐adjusted life expectancy (QALE) of 0.07. They do not report separate mortality elasticities.

For Italy, to the best of our knowledge, the only causal evidence is provided by Depalo (2019) who uses similar regional data to estimate effect bounds of PdR on total (not amenable) mortality and hospitalization rates. He relies on milder nonparametric assumptions than the common trend assumption required for difference‐in‐differences (DID) to identify the treatment effect bounds. The estimates suggest negative consequences on health indicators, namely hospitalization rates and total mortality rates, but only in some regions.

Our contribution to this literature is twofold. First, we exploit an austerity intervention that was specifically targeted at healthcare expenditure and at certain regions as an instrument, while most of the existing subnational studies use regional data—sometimes in periods of austerity—but IVs that do not rely on differential implementation of austerity measures across time and place. Second, we estimate the effect of the austerity policy only on causes of mortality that are considered amenable to medical intervention, which is the subset of mortality that is likely to be most sensitive to spending variation and therefore a better measure to evaluate health consequences. Indeed, amenable mortality has gained prominence as an indicator of healthcare system performance and as a tool for the analysis of the effects of health policies (Eurostat, 2018; Organisation for Economic Co‐operation and Development [OECD], 2018). For example, Moreno‐Serra and Wagstaff (2010) and Wubulihasimu et al. (2016) have used amenable mortality to study the effects of hospital payment reforms.

The cost containment/austerity reform enacted in Italy since 2008—PdR(financial recovery plan, PdR henceforth)—provides a unique opportunity to compare health effects in districts with and without spending cuts. It was imposed by the central government on Italian regions, but did not affect all of the regions, not at the same time and not to the same extent. And while the regional exposure to this reform was not random but dictated by the degree of budgetary deficits incurred, this natural experiment still allowed for an evaluation of the consequences of spending cuts.

The overall effect of this reform has been a sizable decrease in the national health budget deficit, from 6 billion in 2006 to a bit over 1 billion in 2014, with a considerable reduction in the average annual contribution to national healthcare system deficit of the regions with PdR, from 72.3% in 2006 to 43% in 2015 (Ministero Economia e Finanze, 2014). This rather drastic deficit reduction suggests that PdR may have been effective in reaching its financial targets. But, has PdR also had unintended effects on health and healthcare outcomes? The aim of this study is to test whether, at the margin, health spending affects health and healthcare outcomes. The Italian healthcare system reform is used as an instrument to assess causal effects, although its effects on spending are also of interest in their own right.

We exploit the staggered rollout of recovery plans across regions in Italy between 2004 and 2014 and use an IV approach coupled with two‐way fixed effects for region and time to identify the causal effects of healthcare spending on healthcare provision and quality/health and healthcare outcomes.

We find that—on average—PdR led to annual spending cuts of 3.8%, which in turn resulted—again on average—in a 3% rise in avoidable deaths among both men and women. Cause‐specific estimates suggest that most of these deaths were cancer‐related. These harmful effects on mortality appear to have been mediated by substantial capacity cuts in hospital beds (−6.5%) and health workforce employment (−4%) which, in turn, have led to reductions in the rate of hospitalization (−8.5%), as well as a rise in hospital care seeking in non‐PdR regions, mainly in the north of the country.

The remainder of the study is organized as follows: Section 2 explains the implementation of the recovery plans (PdR) and their timelines. Section 3 describes data and empirical approach. Section 4 presents the results. While, Section 5 summarizes and concludes.

## 2 INSTITUTIONAL SETTING: PdR

In 2001, the Italian constitutional reform led to the decentralization and federalization of the healthcare system, granting almost complete administrative and financial autonomy to each of the 21 regions and autonomous provinces. Since then, healthcare funding targets are fixed by the Ministry of Health and modulated at the regional level in accordance with regional planning targets. However, relatively soon after the federalization, 10 out of 21 regions, due to weak managerial capacity and lower health service performance, failed to reach the set goals and the regional health budgets quickly ran into severe deficits. Therefore, recent history of healthcare expenditure is marked by attempts to place stricter control over regions' health spending.

Since 2006, a process of recentralization has been underway, with a new player intervening in the healthcare system: the Ministry of Finance. In particular, the central government has prompted the defaulting regions to adopt financial recovery plans (PdR) in the form of agreements between the region and the central government. Such agreements constitute a formal commitment from the regions with budget deficit toward the Ministry of Health and the Ministry of Finance to design and implement consolidation path. The ministries became actively involved in designing and approving healthcare services delivery in regions with budget deficits, as well as in the evaluation of regional health services through ex ante and ex post monitoring (Depalo, 2019). The aim of such agreements was to restore financial stability and address structural organizational failures (Ministero della Salute, 2018).

However, PdR schemes were not implemented homogenously in all regions. Two main periods of implementation can be distinguished, that is, since 2007 (in the regions Lazio, Abruzzo, Liguria, Campania, Molise, Sicilia, and Sardegna) and since 2010 (in Calabria, Piemonte, and Puglia). Table 1 provides an overview of policy implementation dates per region.

**TABLE 1:** Overview of signature and entry into force of RP per region

Region	Date of signature	Resolution of approval
Lazio	February 28, 2007	DGR n. 149—March 6, 2007
Abruzzo	March 6, 2007	DGR n. 224—March 13, 2007
Liguria	March 6, 2007	DGR n. 243—March 9, 2007
Campania	March 13, 2007	DGR n. 460—March 20, 2007
Molise	March 27, 2007	DGR n. 362—March 30, 2007
Sicilia	July 31, 2007	DGR n. 312—August 1, 2007
Sardegna	July 31, 2007	DGR n. 30/33—August 2, 2007
Calabria	December 17, 2009	DGR n. 908/09—December 23, 2009
Piemonte	July 29, 2010	DGR n. 1–415—August 2, 2010
Puglia	November 29, 2010	DGR n. 2624—November 30, 2010

*Source*: Ministero della Salute (2018).

Moreover, for those regions that were not administratively capable to achieve the goals set for the first year of implementation, the national government decided to appoint an (external) commissioner in order to pursue the central government's targets (Ministero Economia e Finanze, 2009). The regions commissioned were Lazio, Abruzzo, Campania, Molise, and Calabria. Notably, the external commissioner acted as an agent on behalf of the central government for the implementation and rollout of PdR and for the attainment of its objectives. As a result, the regions with a commissioner lost decision‐making power on healthcare‐related decisions, particularly on those decisions relevant for the achievement of PdR objectives. Despite the heterogeneity both in the type and timing of PdR implementation, several common elements of spending cuts and reduction of resources use can be identified among regions with PdR against regions without PdR (Aimone Gigio et al., 2017). These characteristics are the focus of the present analysis to assess the effects of the overall PdR policy on health outcomes, while accounting and adjusting for inter‐regional variances. Therefore, and for the purpose of this analysis, the 20 Italian regions can be divided into two groups: 10 regions without PdR (regions that were never subjected to any PdR) and 10 regions with PdR.

Overall, PdR can be defined as plans for restructuring aimed at affecting the cost factors that led to the drastic increase of regional healthcare budget deficits. There were mainly six areas of cost reduction actions: (i) hospital network reorganization: focused on reduction in hospital beds and incentivization of de‐hospitalization; (ii) pharmaceutical consumption management: control over direct pharmaceutical distribution and implementation of reimbursement mechanisms for less expensive drugs; (iii) healthcare system workforce reorganization: hiring stop and freezing of turn‐over; (iv) accredited private sector management: reducing volumes of services provided by—and the number of—accredited private facilities; (v) centralized purchasing: centralization of purchasing and close monitoring of purchasing to avoid increases in level of spending; and (vi) appropriateness of prescription: utilization of health insurance card system for the improvement of prescription behavior and appropriateness (Ministero Economia e Finanze, 2009).

The regional spending cuts have not passed unnoticed. Throughout the years of implementation, PdRs have received societal attention and media coverage. For example, a recent article analyzed the long‐term effects of PdRs over the last 8 years in Campania (a southern Italian region; Gaita, 2019). It reported the effects of over 45,000 units cut in workforce, closure of hospitals, and severe shortages in hospital beds availability. Similar patterns have been reported in Puglia and Calabria (Marino, 2016). All three are southern Italian regions under PdR.

Of particular concern is that PdRs may have exacerbated geographical health inequalities. Unequal cuts in health spending per capita may have led to a decrease in the volume of healthcare facilities and services available, which may have in turn resulted in worsened population health outcomes. While evidence suggests that fiscal decentralization helped to reduce inequalities within regions (Di Novi, Piacenza, Robone, & Turati, 2019), the consequences of PdR policies are particularly relevant with respect to between‐region disparities which have persisted for decades despite allocation formulas aimed at equalizing the distribution of financial resources (Ferré et al., 2014; Ricciardi, Belvis, Marino, Santoro, & Silenzi, 2011).

The availability of amenable mortality statistics at the regional level has been argued to be a powerful indicator of regional health sector performance (Fantini et al., 2012). As our main health outcomes, we will analyze regional amenable mortalities, defined as preventable and treatable causes of deaths. We will first test whether decreasing healthcare financing and provision has had a positive marginal effect on amenable mortality rates. We then extend the concept to include healthcare utilization and an index of migration. As people in Italy can access medical care regardless of their region of residence, we anticipate that PdRs may also have influenced the utilization and inter‐regional migration patterns of patients, as suggested in a recent national newspaper article (Russo, 2019).

## 3 DATA AND METHODS

Our data were obtained from the Italian National Institute of Statistics (ISTAT) through the software “Health for All”, which is publicly available on the ISTAT website (Istituto Nazionale di Statistica [ISTAT], 2019). We have assembled a panel data set for all variables for all 20 regions for 11 years (2004–2014). As a result, we use variation across 220 observations of regional health spending rates and other covariates.

### 3.1 Avoidable mortality by region

The concept of avoidable mortality, denoting a subset of causes of mortality that are potentially preventable given effective and timely health care, has been developed and analyzed by different authors, including Nolte and McKee (2003, 2004, 2008) and Tobias and Yeh (2009). However, in line with previous publications on amenable mortality in Europe (Mackenbach et al., 2017) and Italy (Fantini et al., 2012), in this study, we use the list of amenable mortality causes for certain age groups developed by Nolte and McKee, (2004).

From the full list of amenable mortalities developed by Nolte and McKee, we could only use the cause‐specific rates of amenable mortality for which regional data for the period 2004–2014 are available from ISTAT. The list of included causes of amenable mortality is presented in Table 2. For most causes, avoidable mortality is restricted to those under 75, but for some causes, other age limits apply. All rates of amenable mortality included were also aggregated to population‐weighted averages standardized by age and gender stratified (male mortality and female mortality). Furthermore, to obtain disease‐specific estimates, we also examine amenable mortalities disaggregated into five disease areas: circulatory system, neoplasms, infectious diseases, respiratory system, and diabetes.

**TABLE 2:** Description of outcome variables

Total amenable mortality (ICD‐10 code)	Disease‐area mortality (ICD‐10 code)	Specific mortality (ICD‐10 code)	Description	Mean 2004	Mean 2014
Female mortality (A15–A19; J10–J18; C18–C21; C50; C53–C55; E10–E14; I20–I25; I60–I69; J00–J99 excl. J10–J18)	Infectious (A15–A19; J10–J18)	Tuberculosis (A15–A19)	Age‐standardized mortality rate for tuberculosis per 10,000 females	0.010	0.012
		Pneumonia and influenza (J10–J18)	Age‐standardized mortality rate for pneumonia and influenza per 10,000 females	0.011	0.099
	Neoplasms (C18–C21; C50; C53–C55)	Colorectal (C18–C21)	Age‐standardized mortality rate for colon, rectal, and anal Cancer per 10,000 females	1.071	0.908
		Breast (C50)	Age‐standardized mortality rate for breast cancer per 10,000 females	2.084	1.893
		Cervical and uterus (C53–C55)	Age‐standardized mortality rate for cervical and uterus cancer per 10,000 females (< 45 years)	0.042	0.046
	Diabetes (E10–E14)	Diabetes (E10–E14)	Age‐standardized mortality rate for diabetes mellitus per 10,000 females(< 45 years)	0.008	0.010
	Circulatory (I20–I25; I60–I69)	Ischemic (I20–I25)	50% of age‐standardized mortality rate for heart diseases per 10,000 females	1.731	1.053
		Cerebrovascular (I60‐I69)	Age‐standardized mortality rate for cerebrovascular diseases per 10,000females	1.294	0.907
	Respiratory (J00–J99 excl. J10–J18)	Respiratory (J00–J99 excl. J10–J18)	Age‐standardized mortality rate for all respiratory disease (excl. pneumonia/influenza) per 10,000 females (0–14 years)	0.110	0.049
Total female amenable mortality			Age‐standardized mortality rate per 10,000 females	6.364	4.934
Male Mortality (A15–A19; J10–J18; C18–C21; C50; C53–C55; E10–E14; I20–I25; I60–I69; J00–J99 excl. J10–J18)	Infectious (A15–A19; J10–J18)	Tuberculosis (A15–A19)	Age‐standardized mortality rate for tuberculosis per 10,000 males	0.035	0.026
		Pneumonia and influenza (J10–J18)	Age‐standardized mortality rate for pneumonia and influenza per 10,000 males	0.201	0.188
	Neoplasms (C18–C21; C50; C53–C55)	Colorectal (C18–C21)	Age‐standardized mortality rate for colon, rectal, and anal cancer per 10,000 males	0.741	0.609
	Diabetes (E10–E14)	Diabetes (E10–E14)	Age‐standardized mortality rate for diabetes mellitus per 10,000 males (< 45 years)	0.019	0.023
	Circulatory (I20–I25; I60–I69)	Ischemic (I20–I25)	50% of age‐standardized mortality rate for ischemic heart diseases per 10,000 males	5.148	3.449
		Cerebrovascular (I60–I69)	Age‐standardized mortality rate for cerebrovascular diseases per 10,000 males	2.008	1.462
	Respiratory (J00–J99 excl. J10–J18)	Respiratory (J00–J99 excl. J10–J18)	Age‐standardized mortality rate for all respiratory disease (excl. pneumonia/influenza) per 10,000 males (1–14 years)	0.122	0.044
Total male amenable mortality			Age‐standardized mortality rate per 10,000 males	9.195	6.604
Expenditure			Regional per‐capita public healthcare expenditure	1550.95	1852.50
Beds			Ordinary hospital bed rate per 10,000 people	38.530	32.685
Employees			Healthcare system employee (workforce) rate per 10,000 people	120.320	114.704
Hospitalizations ALL			Hospitalization rate for any condition per 1000 people	138.102	116.648
Hospitalizations CD			Hospitalization rate for cardiovascular diseases per 10,000 people	2639.88	2009.88
Hospitalizations T			Hospitalization rate for tumors per 10,000 people	1356.29	1147.15
X border:			Index of migration (% of migrants) to other region for hospitalization	10.105	10.685

*Note*: Age limit is 75 years except if otherwise mentioned.

*Source*: ISTAT (National Institute of Statistics).

### 3.2 Other regional outcome variables

As indicators of regional healthcare resources, we use data on regional per capita public healthcare expenditure (expenditure), ordinary hospital bed rate (beds), and healthcare employee rate (employees).

Public healthcare expenditure accounts for the great majority of total healthcare expenditure in Italy, from 75% in 2001 to 80% in 2009 and down to 76.8% of 2014. Overall, private healthcare expenditure never accounted for more than 25% of the total healthcare expenditure (ISTAT, 2019) and mainly consisted of out‐of‐pocket expenditure (Ferré et al., 2014).

As measures of regional healthcare utilization, we use the hospitalization rate for any condition (hospitalizations ALL), hospitalization rate for cardiovascular diseases (hospitalizations CD), hospitalization rate for cancers/tumors (hospitalizations T), and cross‐regional border hospital care seeking (X border).

The rate of cross‐regional border (hospital) care seeking behavior (i.e., the percentage of patients seeking hospital care in another region) is particularly relevant in the context of the federal Italian healthcare system. It measures of the extent to which patients seek hospital care in another region—primarily residents from southern regions going to northern region hospitals. While the patients bear the cost of going there, the cost of the cross‐border care delivered is still to be paid for by the home region.

Finally, we use a set of control variables at the regional level, including population size registered on January 1, average disposable income per capita and GDP per capita, share of population above 64 years old, and young dependency ratio.

### 3.3 Effect identification

The identification of the causal effect of cost containment on avoidable mortality requires us to address two possible sources of endogeneity. First, there may be reverse causality if regions with higher rates of mortality also historically show higher rates of expenditure. Second, there may be unobserved heterogeneity across regions. It is likely that some of the unobserved differences between treated and control regions also may vary over time. In that case, even ordinary least squares (OLS) estimation of a two‐way fixed effects regression will not yield unbiased estimates. One could think of health policies and healthcare practices that influence both public health expenditure and avoidable mortality. To control for this time‐varying heterogeneity and deal with the endogeneity deriving from it, we prefer to go beyond the standard two‐way fixed effects approach often used in this literature (as e.g., in Ruhm's studies) and exploit the effect of PdR as an exogenous change on public health expenditure as an instrument in identifying the spending effect on avoidable mortality.

Thus, in order to estimate the causal effect of cost containment induced by the recovery plans (PdRs) on rates of amenable mortality, we combine an IV approach with a two‐way fixed effects model. We exploit the temporal and geographical variation induced by the administrative changes due to the policy rollout as an IV in a standard two‐stage least squares approach. We estimate the following first‐stage equation:

(1) $${\text{Exp}}_{rt}=\alpha +\sum_{k}\beta_{k}{\text{PdR}}_{rk}+\delta X_{rt}+\gamma_{r}+\mu_{t}+\varepsilon_{rt}$$

where ${\text{Exp}}_{rt}$ is the per capita health expenditure in region *r* in time *t*. PdR is an indicator which takes the value 1 if the region *r* is under PdR in each time period *k*, and the value 0 otherwise. $X_{rt}$ is a vector of control variables at regional level including population size (recorded on January 1), pro‐capita disposable income and GDP per capita. $\gamma_{r}$ and $\mu_{t}$ are region‐ and time‐fixed effects. The region‐fixed effects capture the unobserved regional heterogeneity that is time‐invariant while the time‐fixed effects capture the time trend that is common across regions. The predicted values of expenditure $\hat{{\text{Exp}}_{rt}}$ derived from Equation (1) are then used in the second‐stage regression equation as follows:

(2) $$\text{log}{\left(Y\right)}_{rt}=\alpha +\beta \hat{{\text{Exp}}_{rt}}+\delta X_{rt}+\gamma_{r}+\mu_{t}+\varepsilon_{rt}$$

where *Y*_*rt*_ is the population health outcome of interest observed in region *r* in time *t*. *β* is our coefficient of interest and captures the effect of expenditure changes on health outcomes.

The rationale behind making use of PdR as an instrument deserves some further discussion. There are two main conditions to hold for an instrument to be reliable. First, the IV must be valid, which means that the instrument cannot be correlated with the error term in the second stage. In other words, PdR may only affect the outcome through expenditure, not through any other route. In our data, the correlation between the PdR dummy and amenable mortality is actually very low, being −0.04 for males and −0.02 for females, respectively. This increases the confidence in the assumption that expenditure is the only indirect channel between the two. In fact, as highlighted above, the main aim of the PdR implementation was to restore financial stability of the regions. It was a national policy imposed on the regions running the larger deficits. It directly affected the regional per capita health expenditure resulting from austerity measures that included the reduction of the regional hospital bed capacity and medical human resources. As such, it identifies plausibly exogenous variation in health expenditure.

Second, it must be powerful. In this case, the instrument is highly correlated with the expenditure per capita because the main consequence of PdRs rollout was to contain costs and de facto reducing the health expenditure. Thus, PdRs are expected to explain much of the observed variation across time and region in expenditure. Moreover, we check for the power of the instrument in the first stage using the *F*‐test on whether the excluded instrument significantly differs from zero (Staiger and Stock, 1997). These values are substantially above both the rough, but commonly used, rule of the thumb of 10 and the more formal cutoffs provided by Stock and Yogo (2005).

Using PdR in an IV framework allows us to make our contribution threefold. In the first stage, we are able to estimate the average impact of the policy intervention on the regional healthcare spending. In the second stage, we exploit this variation to estimate the health consequences at the margin of the budget cuts. Third, we employ a similar IV strategy to examine potential mechanisms of the austerity measures, by estimating the expenditure effect on intermediate outcomes like hospital beds per capita and other measures of regional healthcare capacity and utilization.

## 4 RESULTS

This section presents the main results of our empirical analysis in four steps. First, we provide some descriptive findings through a graphical analysis. Second, we document the intended effect of PdR in terms of cuts in expenditure. Third, we estimate the effect of austerity in healthcare on overall amenable mortality and then for the distinct causes of mortality, separately for males and females. Finally, we estimate expenditure effects on intermediate health care outcomes like hospital capacity and utilization as potential pathways that help explaining these results.

### 4.1 Graphical analysis

**FIGURE 1:**
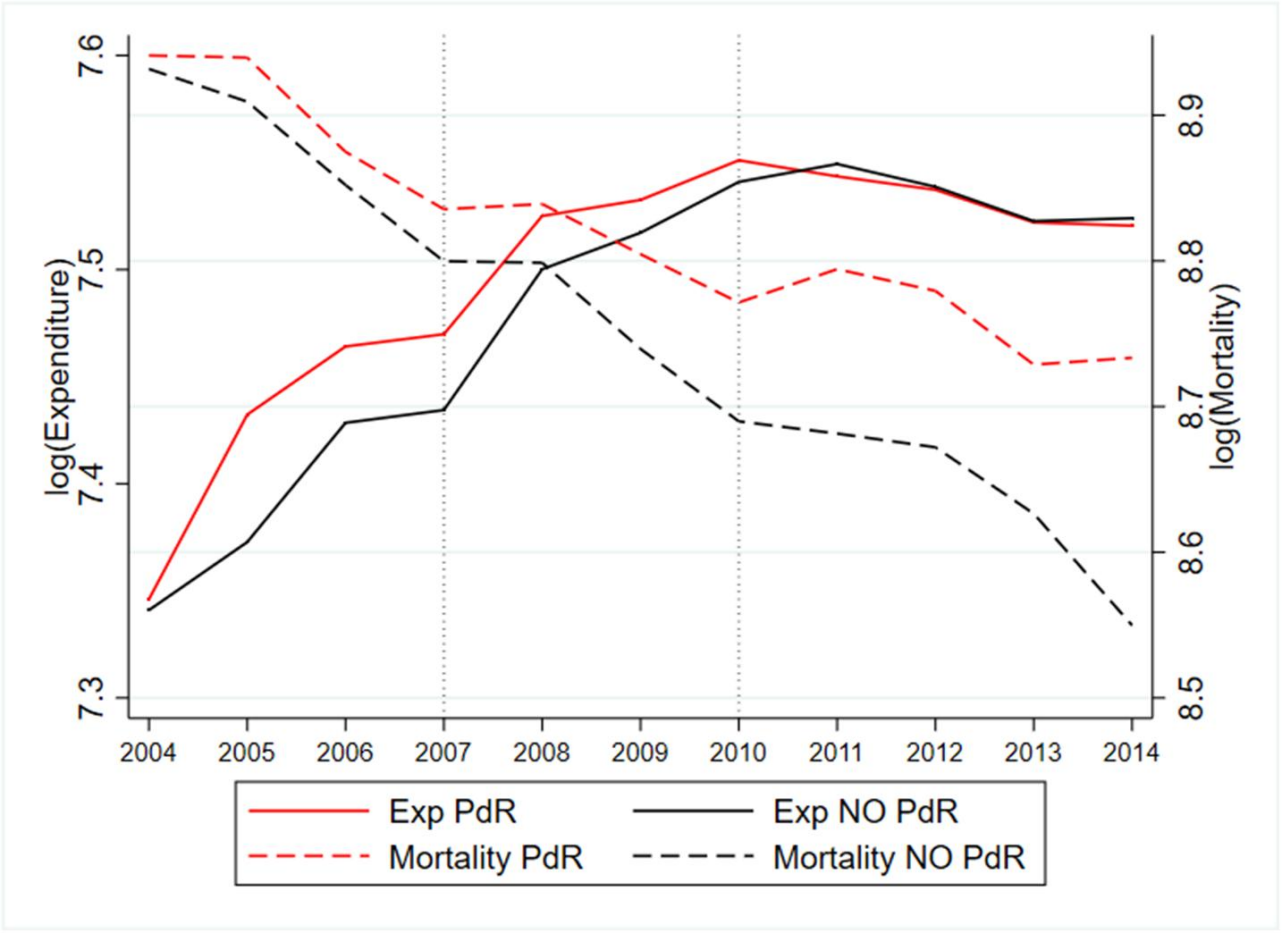
Time trends in (logs of) healthcare expenditure per capita and overall avoidable mortality: Piano di Rientro (PdR) and non‐PdR regions. The figure shows the time trends of the logarithm of healthcare expenditure per capita (left axis) and the logarithm of total amenable mortality (right axis). The two vertical dotted lines depict the two periods of PdR rollout [Colour figure can be viewed at wileyonlinelibrary.com]

**FIGURE 2:**
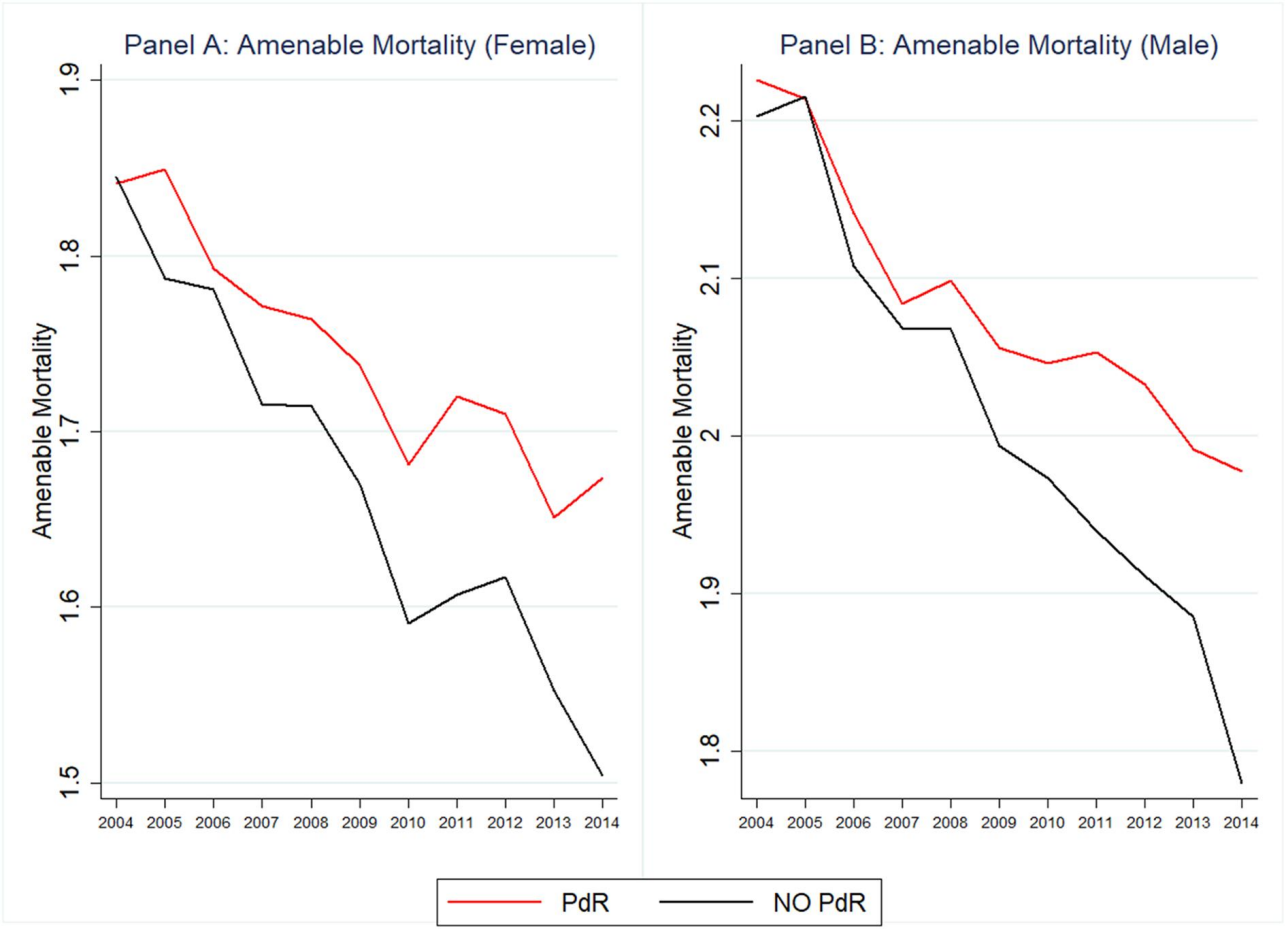
Time trends in (logs of) female and male avoidable mortality: Piano di Rientro (PdR) and non‐PdR regions. The figure shows the time trends for the logarithm of total amenable mortality for female (a) and male (b) [Colour figure can be viewed at wileyonlinelibrary.com]

Figure 1 displays the trends for healthcare expenditure per capita and mortality across PdR (red lines) and non‐PdR regions (black lines). It clearly shows that after the gradual PdR rollout in 2007, 2009, and 2010, there was a closing of the expenditure gap across regions. However, on the other side, this was accompanied by a widening of the mortality gap between PdR and non‐PdR regions, with the gap gradually becoming wider over time. The same patterns emerge for both female as for male mortality, which are displayed in Figure 2.^1^

### 4.2 Model estimates

The first‐stage regression results in Table 3 show that the implementation of PdR has led to a significant reduction in per capita expenditure of about €68, corresponding to a drop of about 3.8%. This is likely to represent the intended effects of the policy, which was aimed at restoring the financial stability through budget cuts. While the €68 annual reduction may seem small compared to the average budget deficits running up to 7%–10% annually, we will examine next whether they may have had any unintended effects on mortality that was potentially avoidable.

**TABLE 3:** First‐stage estimates

	(1)	(2)
	Expenditure	Ln(Expenditure)
PdR	−68.052^***^ (*14.250*)	−0.038^***^ (*0.008*)
Controls	Yes	Yes
Year FE	Yes	Yes
Region FE	Yes	Yes
Obs.	220	220

*Notes*: Parameter estimates of Equation (1). Controls include population size recorded on January 1, pro‐capita disposable income and GDP. Standard errors in *italics*.

Abbreviation: PdR, piano di rientro.

***, **, * indicate statistical significance at 1%, 5%, and 10%, respectively.

Table 4 shows the estimates of our baseline IV model with two‐way fixed effects (years and regions) as well as some time‐varying controls like population size, income per capita, and GDP. Columns (1) and (2) report the estimates of the effect of health expenditure on total amenable mortality for males and females, respectively. Columns (3) and (4) report the same effect in terms of elasticity.

**TABLE 4:** Estimates of the effect on overall amenable mortality by sex

	(1)	(2)	(3)	(4)
	Male mortality	Female mortality	Male mortality	Female mortality
	IV	IV	IV‐elasticity	IV‐elasticity
Expenditure	−0.00044^**^ (*0.0002*)	−0.00045^**^ (*0.0002*)	‐	‐
Ln (expenditure)	‐	‐	−0.785^**^ (*0.363*)	−0.802^**^ (*0.388*)
Controls	Yes	Yes	Yes	Yes
Year FE	Yes	Yes	Yes	Yes
Region FE	Yes	Yes	Yes	Yes
First stage *F*‐test	*28.111*	*28.111*	*27.694*	*27.694*
Obs.	220	220	220	220

*Notes*: Parameter estimates of Equation (2). Dependent variable is the logarithm of amenable mortality. Controls include population size, per capita disposable income and GDP per capita, share of population above 64 years old, and young dependency ratio. Standard errors in *italics*.

***, **, * indicate statistical significance at 1%, 5%, and 10%, respectively.

We find that a decrease of €100 in per capita expenditure on health caused a 4.5% rise in amenable mortality, which is consistent for both males and females. In terms of elasticity, we estimate an elasticity of amenable mortality with respect to health expenditure of approximately −0.8. In other words, the average 3.8% spending cut resulted in a 3% rise in the avoidable death rates. All these estimates are fairly precise and statistically significantly different from 0 at the 5% level.

Next, we examine the disaggregated effects of austerity in healthcare by cause‐specific mortality, stratified by sex. Table 5 displays parameter estimates for males (panel A) and females (panel B), respectively.

**TABLE 5:** Estimates of the effect on amenable mortality by cause and sex

	(1)	(2)	(3)	(4)	(5)
	Circulatory	Neoplasms	Infectious	Respiratory	Diabetes
Panel A: Males					
Expenditure	−0.00048^**^ (*0.0002*)	−0.00234^***^ (*0.0008*)	−0.00012 (*0.0011*)	−0.00211 (*0.0024*)	0.00108 (*0.0022*)
Panel B: Females					
Expenditure	−0.00034 (*0.0003*)	−0.00087^**^ (*0.0004*)	−0.00114 (*0.0012*)	−0.00238 (*0.0033*)	0.00302 (*0.0019*)
Controls	Yes	Yes	Yes	Yes	Yes
Year FE	Yes	Yes	Yes	Yes	Yes
Region FE	Yes	Yes	Yes	Yes	Yes
Obs.	220	220	220	220	220

*Notes*: Parameter estimates of Equation (2). Dependent variable is the logarithm of amenable mortality by cause. Controls include population size recorded on January 1, per capita disposable income and GDP per capita, share of population above 64 years old, and young dependency ratio. Standard errors in italics.

***, **, * indicate statistical significance at 1%, 5%, and 10%, respectively.

Concerning male mortalities, we find a negative and statistically significant effect for circulatory system amenable mortality and neoplasms amenable mortality (mainly due to colorectal cancer for men), with a coefficient −0.0005 and −0.002, respectively. The analysis also reveals negative coefficients for infectious disease mortality (column 3) and respiratory diseases mortality (column 4). However, in both cases, the results are not statistically significant at conventional levels. For females, we find a negative, and statistically significant, effect only on neoplasms amenable mortalities (i.e., mainly breast and cervical cancers).

Finally, we explore some potential pathways for the mortality effects we find. Table 6 displays estimates of the effect of cost containment, induced by PdR schemes, on several measures of healthcare resources and utilization: hospitalizations (columns 1–3), healthcare‐related migration (column 4), hospital bed rates (column 5), and healthcare employment (column 6).

**TABLE 6:** Estimates of the effect on health‐care utilization and resources

	(1)	(2)	(3)	(4)	(5)	(6)
	Hospitalizations ALL	Hosp. CD	Hosp. T	X Border	Beds	Employees
	IV	IV	IV	IV	IV	IV
Expenditure	0.00137^***^ (*0.0003*)	0.00035^**^ (*0.0002*)	0.00025 (*0.0002*)	−0.00074^**^ (*0.0003*)	0.00097^***^ (*0.0003*)	0.0005^**^ (*0.0002*)
Controls	Yes	Yes	Yes	Yes	Yes	Yes
Year FE	Yes	Yes	Yes	Yes	Yes	Yes
Region FE	Yes	Yes	Yes	Yes	Yes	Yes
First stage *F*‐test	*28.111*	*28.111*	*28.111*	*28.111*	*28.111*	*28.111*
Obs.	220	220	220	220	200	200

*Notes*: Parameter estimates of Equation (2). Dependent variable is the logarithm of utilization outcome. Controls include population size recorded on January 1, per capita disposable income and GDP per capita, share of population above 64 years old, and young dependency ratio. Standard errors in italics.

***, **, * indicate statistical significance at 1%, 5%, and 10%, respectively.

Overall, hospitalizations show a reduction of about 13% for a €100 change in per capita expenditure, which is statistically significant at 1% level. For the cause‐specific hospitalizations, we find a significant 3.5% drop in those for cardiovascular diseases and 2% with respect to tumors. However, the latter is not statistically significant.

With respect to the healthcare‐related cross‐border migration, column (4) shows that the austerity has had a strong and statistically significant effect on regional border crossing in care seeking, which amounts to a 7% increase for a €100 reduction in per capita expenditure. This result highlights a major concern for both between and within‐region inequality. First, cross‐border care use tends to increase the (travel) access cost for those who seek care from other regions, but the hospital bill is still picked up by the home region. Second, it increases the within‐region access gap between individuals with different ability to pay for seeking treatments in other regions. For equity reasons, both patterns seem undesirable and PdR is likely to have further promoted treatment differences that favor those able and willing to travel to other regions to obtain potentially better (or at least quicker) care. In that sense, PdR may have further worsened equity in healthcare access between rich and poor.

Finally, columns (5) and (6) show strong effects on capacity measures like hospital beds and the healthcare system workforce that amount to about 10% and 5%, both of which statistically significant. These findings strongly suggest that the spending cuts were translated mainly into medical capacity reductions in PdR regions that in turn induced hospital utilization drops and patient migration patterns.

### 4.3 Robustness checks

In order to examine the robustness of our results, we perform several checks. First, we investigate the first‐stage linking PdR to expenditure. In particular, we perform a simulation exercise through randomization‐based statistical inference for significance tests. We simulate the effect of PdR by randomly assigning a placebo policy to regions at different points in time, in place of the real one.

**FIGURE 3:**
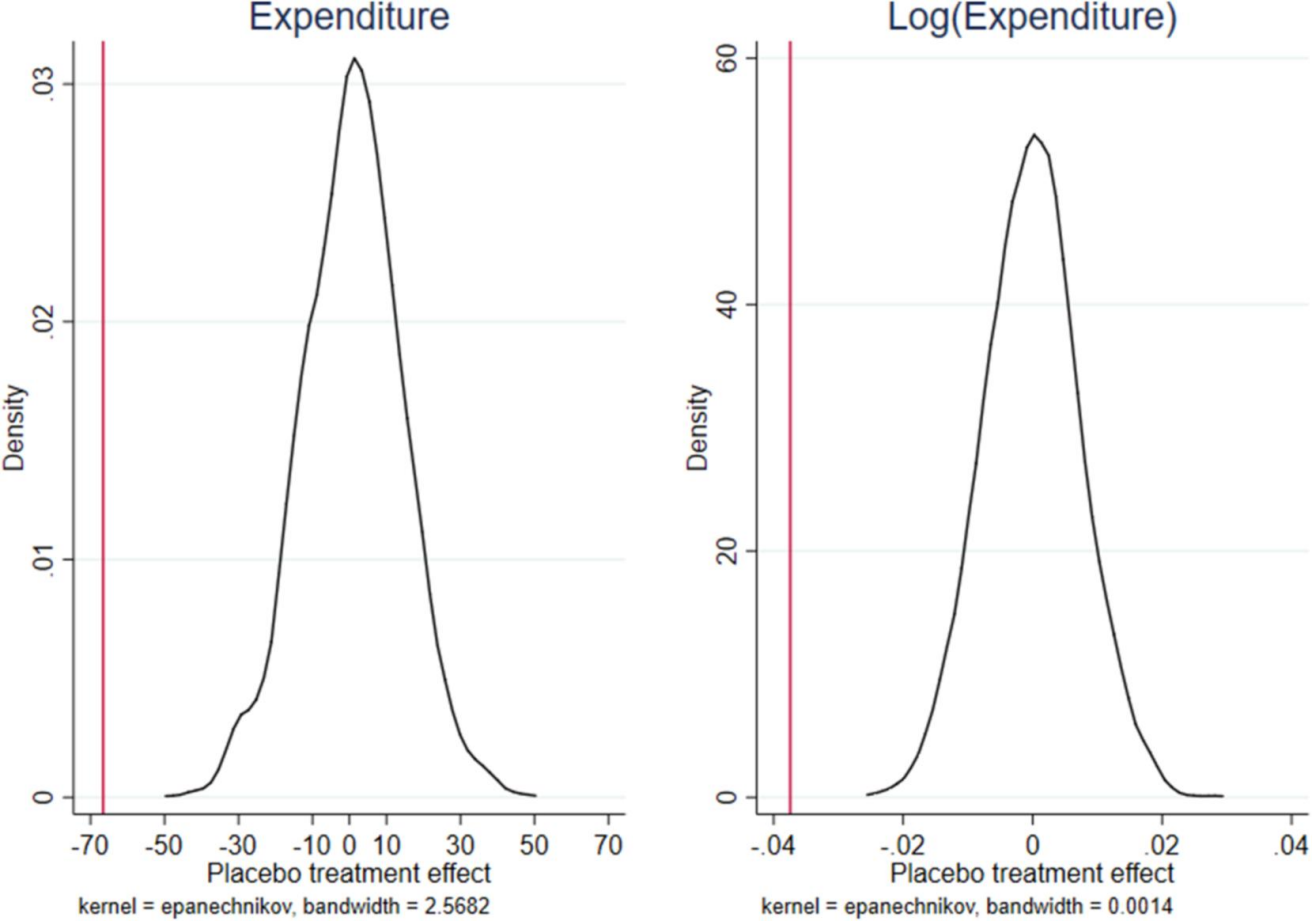
Randomization inference. The figures show the distributions of the placebo estimates based on 2000 permutations for expenditure and log (expenditure), separately. The vertical red lines depict the respective first‐stage coefficients in Table 2 [Colour figure can be viewed at wileyonlinelibrary.com]

We repeat this procedure 2000 times in order to generate a distribution of placebo treatment effects. Figure 3 presents the nonparametric distributions of these placebo estimates for both expenditure and its logarithm transformation, separately.

Figure 3 shows that the average of the placebo treatments is zero and the actual coefficients, which are depicted by the red vertical lines, fall far from the tail of the distribution. This clearly indicates that the negative and significant effect that we find in the first stage is very unlikely to have occurred for reasons other than PdR.

Second, we estimate our model using as dependent variables the overall mortality rates and the nonamenable mortality rates, for both males and females, separately. In fact, by definition, these are not supposed to be affected by the cost containment. Indeed, the results in Table 7 show no significant effect on any of the outcomes considered.

**TABLE 7:** Robustness checks: Overall and nonamenable mortality

	(1)	(2)	(3)	(4)
	Overall male mortality	Overall female mortality	Non‐amenable male mortality	Non‐amenable female mortality
Panel A	IV–Elast.	IV–Elast.	IV–Elast.	IV–Elast.
Log(Expenditure)	−0. 17648 (*0.1429*)	−0. 15038 (*0.1285*)	−0.12872 (*0.1371*)	−0.08460 (*0.1303*)
Controls	Yes	Yes	Yes	Yes
Year FE	Yes	Yes	Yes	Yes
Region FE	Yes	Yes	Yes	Yes
First stage *F*‐test	*27.694*	*27.694*	*27.694*	*27.694*

Panel B	IV	IV	IV	IV
Expenditure	−0.00010 (*0.0001*)	−0.00008 (*0.0001*)	−0.00007 (*0.0001*)	−0.00004 (*0.0001*)
Controls	Yes	Yes	Yes	Yes
Year FE	Yes	Yes	Yes	Yes
Region FE	Yes	Yes	Yes	Yes
First stage *F*‐test	*28.111*	*28.111*	*28.111*	*28.111*
Obs.	220	220	220	220

*Notes*: Parameter estimates of Equation (2). Dependent variable is the logarithm of mortality. Controls include population size recorded on January 1, per capita disposable income and GDP per capita, share of population above 64 years old, and young dependency ratio. Standard errors in italics.

***, **, * indicate statistical significance at 1%, 5%, and 10%, respectively.

However, the point estimates are still of some interest, as they allow us to compare our results to previous findings and thus to check for the plausibility of our estimates. Gallet and Doucouliagos (2017), through a meta‐regression analysis, find that the spending elasticity for overall mortality is about −0.13. The one we estimate here is very close, ranging between −0.17 (males) and −0.15 (females). Concerning the Italian case, these estimates also go in the same direction of those provided by Depalo (2019), who identifies treatment bounds on total mortality for each treated region.

These checks—of the reform timing and the main dependent variable—therefore add to the confidence we have in our main estimates of mortality effects of PdR.

Third, in Table 8, we also perform additional checks with respect to the institutional setting. In columns (1) and (2), we report the estimated coefficients of our model when including in the treatment group only regions in which an external commissioner was appointed. We find larger effects on mortality which are consistent the more severe cuts practiced by the commissioner. In columns (3) and (4), we restrict the time window after the policy in order to rule out any concern about potential confounding from the general spending review passed by Monti's government in 2012.^2^ The results remain basically unaltered.

**TABLE 8:** Robustness checks: Institutional setting

	(1)	(2)	(3)	(4)
	Male mortality	Female mortality	Male mortality	Female mortality
	IV commissioner	IV commissioner	IV shorter time window	IV shorter time window
Expenditure	−0.00070^***^ (*0.0002*)	−0.00061^**^ (*0.0003*)	−0.00042^**^ (*0.0002*)	−0.00041^*^ (*0.0002*)
Controls	Yes	Yes	Yes	Yes
Year FE	Yes	Yes	Yes	Yes
Region FE	Yes	Yes	Yes	Yes
First stage F‐test	*29.595*	*29.595*	*21.009*	*21.009*
Obs.	209	209	180	180

*Notes*: Parameter estimates of Equation (2). Dependent variable is the logarithm of mortality. Controls include population size recorded on January 1, per capita disposable income and GDP per capita, share of population above 64 years old, and young dependency ratio. Standard errors in italics.

***, **, * indicate statistical significance at 1%, 5%, and 10%, respectively.

**FIGURE 4:**
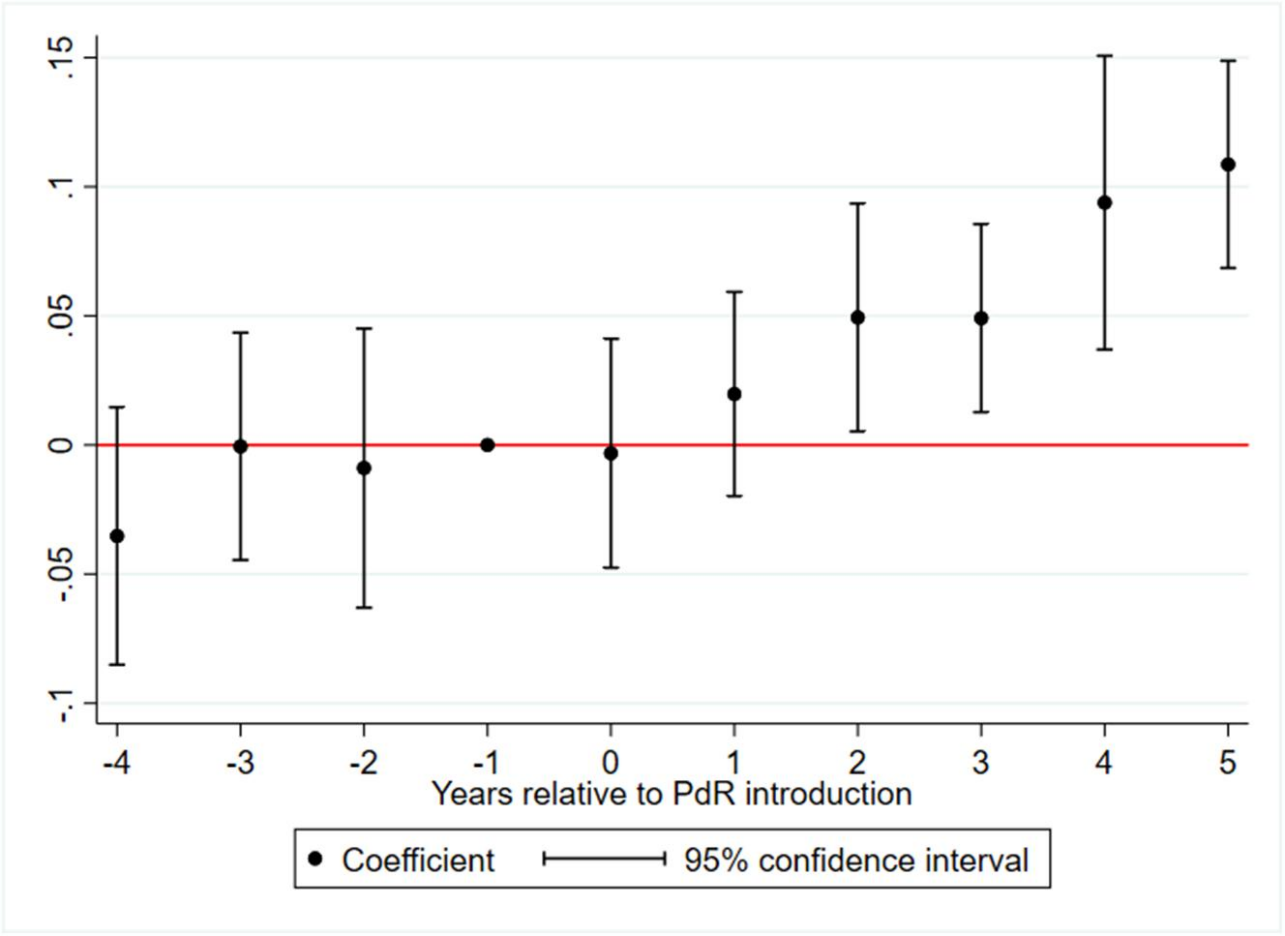
Event study analysis. The figure presents an event study of the effects of Piano di Rientro (PdR) on male avoidable mortality. One‐year pre‐introduction is the excluded dummy for each dimension and is set equal to zero. Similar results are obtained for female avoidable mortality (available upon request) [Colour figure can be viewed at wileyonlinelibrary.com]

Finally, we provide additional evidence on the effect of PdR on amenable mortality when adopting an alternative method using an event study framework. Specifically, we estimate a reduced form of our main model by considering lead and lagged effects of the policy. The results for male mortality are plotted in Figure 4.

These findings support our IV‐based evidence on the effects of PdR as they show a sharp increase in amenable mortality after the policy implementation. Importantly, Figure 4 also removes any possible concerns about systematic pre‐reform trends affecting the results.

## 5 CONCLUSION AND DISCUSSION

We have taken advantage of the natural experiment of the PdR austerity policy that was implemented in selected regions in Italy to revisit an old question in health economics: at the margin, what does additional spending buy in terms of health gains? Interestingly, this was a period of fairly severe cuts in health spending in a relatively high‐income and high‐spending country, where it has often been argued that what is achieved at the margin is not extension of life expectancy but mainly improvement of quality of life. Our findings suggest otherwise.

Confronted with rising health budget deficits, PdR reforms were implemented in 10 of 20 regions—mainly those located in the south of Italy—with the main goal to reduce these deficits by reducing expenditure. We find that—on average—this policy was successful and resulted in annual spending cuts of 3.8% after PdR, or about €68 per capita. But these cuts were not without consequences: they resulted—again on average—in a 3% rise in avoidable deaths among both men and women. Cause‐specific estimates suggest that most of these were cancer related; mainly colorectal cancer and circulatory diseases for males, breast, and cervical cancers for females. These are deaths that were avoidable and that could have been prevented through timely detection and treatment of cases. Robustness checks confirm that these rises only occurred for amenable mortality causes and that they were only due to PdR.

We also find plausible mechanisms for these harmful effects on mortality amenable to intervention. PdR's spending cuts have led to substantial capacity cuts in hospital beds (−6.5%) and health workforce employment (−4%). These may, in turn, have led to reductions in the rate of hospitalization (−8.5%). The spending cuts also seem to have spurred “medical care‐seeking behavior from patients in PdR regions in non‐PdR regions,” as indicated by the rise in (hospital) care seeking in non‐PdR regions. The latter finding causes concern, not so much for health reasons, but for equity reasons: it suggests that those who were able and willing to pay extra to seek care in other—usually northern Italian—regions, have managed to escape the budgetary cuts that may have led to additional health risks. The findings obtained with our IV approach are in line with those of Depalo (2019) but appear to be stronger and more informative because we focus on specific types of amenable mortality and hospitalization.

Overall, we can conclude the following. First, even in rich, high‐spending countries like Italy, budgetary cuts for austerity reasons can lead to substantial loss of life. In Italy, the austerity induced by the fiscal recovery plans has led to cuts that were harmful to health and survival. Second, the reform led to cuts in especially hospital capacity that reduced utilization which in many cases must have been essential use of services, as indicated by the rise in premature deaths of males and females. Especially the rise in some preventable causes of cancer mortality are reasons for concern in an era where many new and very expensive new cancer treatments are knocking on the reimbursement door. Third, it also has had implications for equity within and between regions. Between regions, the gap in spending and mortality has increased, with especially the Southern Italian regions bearing the brunt of the cuts. Within these regions, it is likely to have increased inequity in access as patients that were able and willing to go and seek hospital care in the North of the country.

All in all, PdR seems to have reached its fiscal goals—that is regional budget deficit reductions—at a high price: an increase in preventable deaths, as well as a rise in inequity in healthcare access both within and between regions. In the recent OECD Health at a Glance comparison (OECD, 2019; OECD, 2019), Italy still stands out as a country with a relatively low average spending rate (fourth lowest in the OECD) at $3428 (based on purchasing power parity). However, its 2017 ranking in terms of “treatable mortality” (13th) is not as good as its ranking in terms of “preventable mortality”. The latter category is defined as causes of death that can be mainly avoided through timely and effective healthcare interventions, including secondary prevention and treatment (i.e., after the onset of diseases, to reduce case fatality). It is quite likely that PdR has contributed to this lower ranking of Italy given that its real health expenditure growth rate for the period 2013–2018—a mere 0.8% per year—has lagged far behind the OECD average of 2.4% over the same period. Our results suggest that especially the poorer south of Italy has suffered the survival consequences of this limited expenditure growth.

## CONFLICT OF INTEREST

The authors have no conflict of interests to declare.

## ENDNOTES

^1^ Similar trends are obtained when centering the graph with respect to the PdR introduction, for every region. These are available upon request.

^2^ This fiscal measure was introduced in order to “reduce the public expenditure with unchanged services” (law decree D.L. 95/2012). This was a general national spending review with several areas of intervention. With respect to healthcare main interventions regarded: prescription of generic drugs, expenses for personnel and for procurement of goods, services and medical devices, and ceiling for pharmaceutical expenditure.
